# *Orientia tsutsugamushi* Modulates RIPK3 Cellular Levels but Does Not Inhibit Necroptosis

**DOI:** 10.3390/pathogens14050478

**Published:** 2025-05-14

**Authors:** Thomas E. Siff, Paige E. Allen, David L. Armistead, Jason R. Hunt, Steven J. Rolland, Hervé Agaisse, Jason A. Carlyon

**Affiliations:** 1 Department of Microbiology and Immunology, School of Medicine, Virginia Commonwealth University Medical Center, Richmond, VA 23298, USA; siffte@vcu.edu (T.E.S.); paige.allen@vcuhealth.org (P.E.A.); david.armistead@vcuhealth.org (D.L.A.); jason.hunt@vcuhealth.org (J.R.H.); 2 Department of Microbiology, Immunology, and Cancer Biology, University of Virginia School of Medicine, Charlottesville, VA 22908, USA; yhj2jn@virginia.edu (S.J.R.); hfa5y@virginia.edu (H.A.)

**Keywords:** *Orientia tsutsugamushi*, scrub typhus, obligate intracellular bacterium, *Rickettsia*, necroptosis, RIPK3, MLKL, programmed cell death

## Abstract

Scrub typhus is an emerging chigger-borne disease caused by the obligate intracellular bacterium *Orientia tsutsugamushi*. Necroptosis is a form of programmed cell death (PCD) mediated by RIPK3 (serine/threonine kinase receptor interacting protein 3) and its downstream effector MLKL (mixed-lineage kinase domain-like). While *O. tsutsugamushi* modulates apoptosis, another form of PCD, its interplay with necroptosis is unknown. Much of *Orientia* pathobiology is linked to its ankyrin repeat (AR)-containing effectors (Anks). Two of these, Ank1 and Ank6, share similarities with the cowpox AR protein, vIRD (viral inducer of RIPK3 degradation) that prevents necroptosis. Here, we show that Ank1 and Ank6 reduce RIPK3 cellular levels although not as robustly as and mechanistically distinct from vIRD. *Orientia* infection lowers RIPK3 amounts and does not elicit necroptosis in endothelial cells. In HeLa cells ectopically expressing RIPK3, *Orientia* fails to inhibit RIPK3 and MLKL phosphorylation as well as cell death. MLKL colocalization with *Orientia* or *Listeria monocytogenes*, another intracytoplasmic pathogen, was not observed. Thus, *O. tsutsugamushi* reduces cellular levels of RIPK3 and does not elicit necroptosis but cannot inhibit this PCD pathway once it is induced. This study is a first step toward understanding how the relationship between *Orientia* and necroptosis contributes to scrub typhus pathogenesis.

## 1. Introduction

Programmed cell death (PCD) can limit niches for intracellular pathogens and serve as an initial line of defense especially since pathogen-specific T- and B-cell mediated immunity takes several days to develop [1]. Co-evolutionary arms races between obligate intracellular pathogens and their eukaryotic hosts can select for microbial strategies that avoid eliciting and/or inhibit PCD [1,2,3,4]. Therefore, studying the interplay between PCD and intracellular microorganisms is paramount to better understanding the pathobiology of the diseases they cause. PCD results in a non-lytic morphology that is immunologically silent, as exemplified by apoptosis, or a lytic morphology that is inflammatory [5]. Necroptosis is a tightly regulated lytic form of PCD mediated by serine/threonine kinase receptor interacting protein 3 (RIPK3) and its downstream effector mixed-lineage kinase domain-like (MLKL). Various stimuli lead to RIPK3 autophosphorylation, which enables RIPK3 to phosphorylate MLKL. Phosphorylated MLKL oligomerizes on the plasma membrane to execute lytic cell death and release damage-associated molecular patterns (DAMPs) [1,6,7].

*Orientia tsutsugamushi* is a mite-transmitted obligate intracellular bacterium that replicates to high loads in the cytosol of endothelial cells and leukocytes to cause the emerging spotted fever illness, scrub typhus. This potentially deadly rickettsiosis is prominent in the Asia–Pacific and also occurs in the United Arab Emirates, several African countries, Chile, and the Peruvian Amazon [8,9,10,11,12,13,14,15,16,17]. *Orientia* DNA was recently detected in chiggers at sampling sites in North Carolina [18,19], indicating that scrub typhus might be present in the United States. *O. tsutsugamushi* delays apoptosis of multiple host cell types, and this is functionally linked, at least in part, to several of its ankyrin repeat (AR)-containing effectors (Anks) [20,21,22,23,24]. Whether *Orientia* invokes or modulates necroptosis has not been investigated.

Much of the knowledge into how pathogens inhibit necroptosis has been provided through the study of viruses, especially herpes simplex virus and poxviruses [1,2,3,4]. Notably, poxviral Anks are structurally similar to *O. tsutsugamushi* Anks and, like *Orientia* Anks, play key roles in subverting host immunity [4,23,25,26,27,28,29,30,31,32]. Both types of these microbial Anks have N-terminal AR and C-terminal F-box domains, the latter of which nucleates the SCF (Skp1/cullin-1/F-box) E3 ubiquitin ligase complex that ubiquitinates proteins for 26S proteasomal degradation [26,28,29,30,31,32,33]. Cowpox virus uses an Ank dubbed vIRD (viral inducer of RIPK3 degradation) to rid host cells of RIPK3. vIRD uses its AR domain to bind RIPK3 and its F-box to promote RIPK3 ubiquitination and proteasomal degradation. Cowpox in which vIRD has been deleted is unable to degrade RIPK3 and stimulates infected cells to undergo necroptosis [4]. Like many poxviral Anks, vIRD also inhibits nuclear factor kappa-B (NF-κB) accumulation in the nucleus [25,29,30,31,32], a phenomenon recapitulated by *O. tsutsugamushi* Ank1 and Ank6 [27,28,34]. The shared abilities of vIRD, Ank1, and Ank6 to inhibit NF-κB provided the impetus for us to investigate whether they also share the capacity to promote RIPK3 degradation. Furthermore, the lack of information on the interplay between *Orientia* and necroptosis prompted us to examine whether the cytosolic pathogen stimulates and/or modulates this PCD pathway. Overall, we found that *O. tsutsugamushi* reduces RIPK3 levels and does not elicit necroptosis but is unable to inhibit necroptosis once it has been induced.

## 2. Materials and Methods

### 2.1. Cell Cultivation and Bacterial Infections

Uninfected HeLa cells (CCL-2, American Type Culture Collection [ATCC], Manassas, VA, USA) and EA.hy926 cells (CRL-2922, ATCC) were maintained as described previously [26,27]. *Orientia tsutsugamushi* str. Ikeda, which was originally isolated from a scrub typhus patient in Japan [35], was propagated in HeLa cells as described previously [36]. Host cell-free bacteria were obtained for experimental purposes via mechanical lysis of highly infected HeLa cells as described previously [36]. Synchronous infections were performed using a multiplicity of infection (MOI) of 5–10 verified by immunofluorescence assay of coverslips infected in parallel and immunolabeled with antiserum specific for *O. tsutsugamushi* TSA56 (56-kDa type-specific antigen) as described below. Samples had to meet the criterion of having at least 80% of the cells infected to be analyzed further. In some cases, HeLa cells were transfected to express HA-RIPK3 24 h prior to infection or Flag-BAP (*Escherichia coli* bacterial alkaline phosphatase) or Flag-RIPK3 24 h post-infection (hpi). Uninfected HT-29 cells (HTB-38, ATCC) were cultured at 37 °C and 5% CO_2_ in McCoy’s 5A medium (Gibco) supplemented with 10% heat-inactivated fetal bovine serum (Gibco, Waltham, MA, USA). Cells were washed with Dulbecco’s phosphate-buffered saline (DPBS; Gibco), lifted with 0.25% trypsin–EDTA (Gibco), and seeded on glass coverslips in a 24-well plate (Costar, Corning, NY, USA) four days prior to infection. *Listeria monocytogenes* strain 10403S [37] was propagated on brain heart infusion (BHI) agar or in BHI broth. For infection of host cells, an overnight culture of *L. monocytogenes* was centrifuged at 8000 rpm for 1 min and resuspended in DPBS at an optical density (OD) of 0.001 at 600 nm. Diluted bacteria were added to host cell media at a ratio of 100 μL bacteria per mL media and 500 μL of this solution was added to each well of HT-29 cells. The plate was centrifuged at 1,000 rpm for 5 min to initiate and synchronize infection. After 1 h of incubation at 37 °C and 5% CO_2_ to allow bacterial invasion, the media were removed, and cells were washed three times with DPBS. Cell media containing 50 μg/mL gentamicin (Sigma Aldrich G1397, St. Louis, MO, USA) was added to each well to kill extracellular bacteria and the plate was incubated for 5 h at 37 °C and 5% CO_2_ before processing for immunofluorescence microscopy.

### 2.2. Plasmid Constructs

pFlag-BAP was purchased from Sigma-Aldrich (C7472). Constructs encoding N-terminally Flag-tagged Ank1, Ank1∆F-box, Ank6, and Ank6∆F-box were generated previously [27,38]. Mammalian codon-optimized cowpox virus vIRD in the pcDNA-DEST40 plasmid backbone (ThermoFisher, Waltham, MA, USA) was a kind gift from Dr. Grant McFadden (Arizona State University, Tempe, AZ, USA). To generate pFlag-vIRD, flanking 5’ EcoRI and 3’ XbaI restriction sites were added to the vIRD gene via PCR amplification using primers 5’-AATTCTGCAGAATTCAATGAGCACCATCACCAAG-3’ and 5’-GATCAGTTATCTAGACTAGTAGGGGTAGTGCTTGTA-3’. The amplicon was restriction-digested and subcloned into p3XFLAG-CMV-7.1 (Sigma-Aldrich E4026). pcDNA3-HA-RIPK3 (Addgene plasmid #78804; http://n2t.net/addgene:78804; accessed on 4 April 2022; RRID:Addgene_78804) and pcDNA3-FLAG-RIPK3 (Addgene plasmid #78815; http://n2t.net/addgene:78815; accessed on 4 April 2022; RRID:Addgene_78815) were gifts from Dr. Jaewhan Song [39]. All constructs were sequenced to ensure nucleotide fidelity (Genewiz, South Plainfield, NJ, USA).

### 2.3. Transfection

Uninfected or infected HeLa cell cultures grown to at least 90% confluence were transiently transfected with 2–4 μg of plasmid DNA using Lipofectamine 2000 (Invitrogen 11668019, Carlsbad, CA, USA) and OptiMEM I (Gibco 31985-070) per the manufacturer’s protocol. Transfected cells were incubated in a humidified incubator at 37 °C and 5% CO_2_ for 16–24 hours before harvesting or infection with *O. tsutsugamushi*. Spent media were removed and cells were washed with phosphate-buffered saline (PBS; 0.27 mM KCl, 137 mM NaCl, 2 mM KH2PO4, 8 mM Na2HPO4, pH 7.4) before being processed for further applications.

### 2.4. Co-Immunoprecipitation and Western Blot

For infection or transfection studies using whole-cell lysates, cells were washed once with PBS or Tris-buffered saline (TBS; 6.25 mM Tris base, 34.25 mM NaCl, 0.67 mM KCl, pH 7.6), collected by trypsinization or scraping, and lysed in radioimmunoprecipitation assay buffer (RIPA; 50 mM Tris HCl, 150 mM NaCl, 1% sodium deoxycholate, 1 mM EDTA, 1% NP-40, pH 7.4) spiked with 1X HALT Protease and Phosphatase Inhibitor Cocktail (Thermo Scientific 1861281). After 45 min, lysed samples were centrifuged at 16,000× *g* for 10 min at 4 °C and the supernatant concentration was quantified by Bradford assay (Bio-Rad 5000006, Hercules, CA, USA). Lysates were normalized in PBS or RIPA buffer (for experiments involving detection of phosphorylated proteins) and diluted in 2X Laemmli sample buffer (Bio-Rad 1610737) with 5% β-mercaptoethanol (VWR 0482, Radnor, PA, USA) for analysis by Western blot. Anti-Flag affinity agarose immunoprecipitation was performed as described previously using four washes prior to elution with 2X Laemmli-β-mercaptoethanol [28]. Normalized amounts of RIPA lysates or immunoprecipitation inputs/eluates were subjected to SDS-PAGE and Western blot as previously described [26]. Blots were imaged on a ChemiDoc Touch Imaging System (Bio-Rad). ImageLab v6.1 software (Bio-Rad) was used to generate images and perform densitometry. Antibodies used were: rabbit anti-hemagglutinin (HA) (Abcam ab236632, Waltham, MA, USA; 1:1000, BSA), mouse anti-Flag (Sigma F1804, 1:1000, milk), rabbit anti-Flag (Sigma F7425, 1:1000, milk), mouse anti-glyceraldehyde-3-phosphate dehydrogenase (GAPDH) (Santa Cruz sc-365062, Dallas, TX, USA; 1:2000, milk), rabbit anti-MLKL (Cell Signaling 14993, Danvers, MA, USA; 1:1000, BSA), mouse anti-MLKL (Proteintech 66675-1-IG, Rosemont, IL, USA; 1:1000, milk), rabbit anti-MLKL (phospho S358) (Abcam ab187091, 1:1000, BSA), rabbit anti-RIPK1 (Cell Signaling 4926, 1:1000, BSA), rat anti-RIPK3 (Millipore-Sigma MABC1640 clone 1H2; 1:500, milk), rabbit anti-RIPK3 (phospho S227) (Abcam ab209384, 1:1000, BSA), rabbit anti-TSA56 (1:4000, milk) [40], horseradish peroxidase (HRP)-conjugated goat anti-rabbit IgG (Cell Signaling 7074, 1:10,000), HRP-conjugated goat anti-rat IgG (Cell Signaling 7077, 1:10,000), and HRP-conjugated goat anti-mouse IgG (Cell Signaling 7076, 1:10,000).

### 2.5. Immunofluorescence Microscopy

To confirm the MOI for each *O. tsutsugamushi* infection experiment, glass coverslips with infected cells were washed once with PBS and fixed with ice-cold methanol for 5 min at 2–4 hpi. Coverslips were washed twice more with PBS and blocked in 5% (*w*/*v*) bovine serum albumin (BSA) in PBS for 1 h, then incubated with rabbit antisera generated against TSA56 at 1:1000 dilution in 5% BSA-PBS for 1 h. Coverslips were washed twice with PBS and incubated with secondary antibody (1:1000 Alexa Fluor 488-conjugated chicken anti-rabbit IgG [ThermoFisher Scientific A21441]) in the dark for 1 h. Coverslips were washed 3 more times with PBS and mounted using ProLong Gold Anti-fade reagent (Invitrogen P36930). Slides were imaged using a Leica DMi8 inverted microscope affixed with the following Leica package: Leica EL6000 lamp at 460 nm and 630 nm and band-pass filters at 420/30 nm and 570/20 nm. Image acquisition was performed with an Andor iXon Ultra 888 EMCCD camera (Oxford Instruments, Concord, MA, USA) and a 63X water-immersion objective with 1.2 numeric aperture. The average number of bacteria per cell in 30 cells was calculated to determine the MOI. To assess MLKL localization during *O. tsutsugamushi* infection, glass coverslips containing uninfected or infected HeLa cells at 72 hpi were washed once with PBS and fixed in ice-cold methanol for 5 min, then processed using the same protocol. Samples were incubated with rat anti-MLKL (Millipore-Sigma MABC1635 clone 10C2, 1:200) and rabbit anti-TSA56 (1:1000) followed by incubation with Alexa Fluor 488-conjugated chicken anti-rabbit IgG (1:1000) and Alexa Fluor 594-conjugated chicken anti-rat IgG (ThermoFisher Scientific A21471, 1:1000). Confocal micrographs were obtained on a Leica Stellaris 8 laser-scanning confocal microscope using a 63X oil-immersion objective. To assess MLKL localization during *L. monocytogenes* infection, glass coverslips containing uninfected or infected HT-29 cells were washed once in PBS and fixed in ice-cold methanol for 5 min at 6 hpi. Samples were blocked as above and sequentially probed with rat anti-MLKL, Alexa Fluor 594-conjugated chicken anti-rat IgG, unconjugated rabbit anti-*Listeria* (Virostat 4201, Portland, ME, USA; 1:500), and Alexa Fluor 488-conjugated goat anti-rabbit IgG (Invitrogen A11034, 1:1000) to avoid antibody cross-reactivity. Coverslips were obtained on a Leica DMi8 spinning-disc confocal microscope using a 63X oil-immersion objective and equipped with an Andor iXon ULTRA 888BV EMCCD camera and driven by the iQ software (Andor, Concord, MA, USA). Image processing and line-profile analyses were performed using Fiji (https://fiji.sc/; accessed on 11 July 2024) and Imaris x64 software v8.3.1 (BitPlane, South Windsor, CT, USA). The fluorescence intensity values for each channel along the line profile were normalized to the highest and lowest intensity values and plotted in the GraphPad Prism v10.4.1 software package (GraphPad, San Diego, CA, USA).

### 2.6. Necroptosis Induction

To pharmacologically induce necroptosis, uninfected and infected (72 hpi) EA.hy926 cells were pre-treated with 1 mM BV6 (APExBIO B4653, Houston, TX, USA) and 20 mM zVAD-fmk (APExBIO A1902) (BZ) or vehicle (dimethyl sulfoxide [DMSO; Fisher BP231-100]) for 1 h at 35 °C. Media was replaced with fresh media containing 1 mM BV6, 20 mM zVAD-fmk, and 10 ng/mL tumor necrosis factor (TNF; Gibco PHC3015) (TBZ) or vehicle (DMSO and filter-sterilized 0.1% BSA in deionized water). Cultures were incubated at 35 °C for 4 h and assayed by flow cytometry. To induce necroptosis via RIPK3 overexpression, HeLa cells were transfected to express HA-RIPK3 and incubated for 16–24 h at 37 °C. The next day, cultures were inoculated with *O. tsutsugamushi* and incubated in a humidified incubator at 35 °C and 5% CO_2_. Cultures were harvested via RIPA lysis at 24, 48, and 72 hpi and assayed by Western blot. Alternatively, uninfected and infected HeLa cells were transfected to express Flag-RIPK3 or Flag-BAP at 24 hpi. Cultures were incubated at 35 °C for 24 h and assayed by flow cytometry at 48 hpi.

### 2.7. Flow Cytometry

Uninfected and infected EA.hy926 cells were treated with vehicle or TBZ for 4 h then collected and analyzed by propidium iodide (PI)/Annexin V flow cytometry assay (BD Biosciences 556547, Franklin Lakes, NJ, USA) as described [23]. Uninfected or infected HeLa cells were transfected at 24 hpi to express Flag-BAP or Flag-RIPK3 for 24 h before collection and analysis by PI/Annexin V flow cytometry assay. Ten thousand cells per sample were analyzed using a BD FACSMelody with BDFACSChorus 1.3.3 software (BD Biosciences). Compensation and quadrant analysis were performed in FlowJo (version 10.8.1) software (BD Biosciences). Cells positive for PI (quadrant 1 [Q1] [FITC-/PI+] and Q2 [FITC+/PI+]) were considered necroptotic and/or dead. Cells in Q3 (FITC+/PI-) were considered early apoptotic. Cells in Q4 (FITC-/PI-) were deemed to be alive [41].

### 2.8. Statistical Analysis

All statistical comparisons were performed using the GraphPad Prism v10.4.1 software package with α = 0.05. The student’s t-test was used to compare two groups. One-way analysis of variance (ANOVA) with Dunnett’s post hoc test was used to compare two or more groups to a control group. One-way ANOVA with Tukey’s post hoc test was used to compare three or more groups to each other. All error bars represent the mean +/− one standard deviation.

## 3. Results

### 3.1. Ectopically Expressed O. tsutsugamushi Ank1 and Ank6 Reduce RIPK3 Cellular Levels

**Figure 1:**
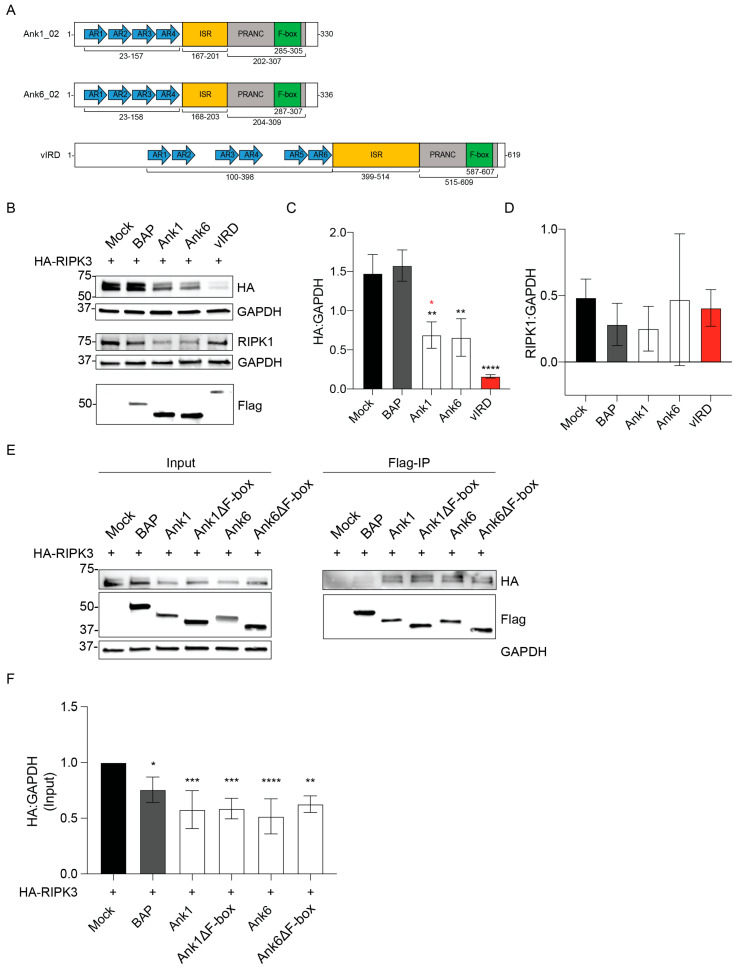
Ank1 and Ank6 bind and lower RIPK3 levels in an F-box-independent manner. (**A**) Schematics of Ank1, Ank6, and vIRD. For Ank1 and Ank6, the number that follows the underscore indicates the specific paralog of the multicopy paralogous family used in this study. Hereafter, the paralog indicator will be excluded. The ankyrin repeats (ARs; blue arrows), intervening sequence region (ISR; orange box), PRANC (pox protein repeats of ankyrin-C-terminal) domain (gray box), and F-box (green box) are indicated, as are the amino acids that encompass each region. (**B**–**F**) HeLa cells were transfected to express HA-RIPK3 and Flag-BAP, the indicated Flag-Ank proteins, or mock-transfected. (**B**) Whole-cell lysates were analyzed by immunoblot with antibodies against HA, Flag, RIPK1, and GAPDH. The HA:GAPDH (**C**) and RIPK1:GAPDH (**D**) densitometric signal ratios per sample were calculated. Asterisks indicate statistical significance compared to Mock + HA-RIPK3 (black) or Flag-vIRD + HA-RIPK3 (red). (**E**) Whole-cell lysates (Input) and eluates following anti-Flag co-immunoprecipitation were analyzed by immunoblot with the indicated antibodies. (**F**) The HA:GAPDH densitometric signal ratio per sample on the input blot was calculated and normalized to Mock + HA-RIPK3. Asterisks indicate statistically significant differences compared to Mock + HA-GAPDH. Results are representative of three (**B**–**D**) or four (**E**,**F**) independent experiments. Data are presented as mean ± standard deviation (**C**,**D**,**F**). One-way ANOVA with Tukey’s (**C**,**D**) or Dunnett’s (**F**) post hoc test was used to test for significant differences between all samples or between Mock + HA-RIPK3 and each other sample, respectively. Statistically significant values are indicated as * *p* < 0.05, ** *p* < 0.01, *** *p* < 0.001, **** *p* < 0.0001.

Because Ank1, Ank6, and vIRD share a bipartite architecture consisting of N-terminal AR and C-terminal F-box domains (Figure 1A) and all three similarly inhibit NF-κB nuclear accumulation [4,25,27,28,29,30,31,32], we initiated this study by testing the hypothesis that Ank1 and Ank6 functionally overlap with the ability of vIRD to reduce RIPK3 cellular levels [4]. To investigate this, we co-transfected HeLa cells to express HA-tagged RIPK3 and Flag-tagged Ank1, Ank6, or vIRD. Negative controls were HA-RIPK3 expressing cells that were also mock transfected or co-transfected to express BAP. Western blot analysis of GAPDH confirmed equivalent loading (Figure 1B). Flag-vIRD nearly depleted HA-RIPK3 levels (Figure 1B,C). Flag-Ank1 and Flag-Ank6 each reduced HA-RIPK3 levels two-fold. While HeLa cells do not endogenously express RIPK3, they do express RIPK1 [42,43]. Flag-Ank1, Flag-Ank6, and Flag-vIRD did not significantly alter RIPK1 levels (Figure 1B,D). Next, Flag-tagged Ank1, Ank6, and C-terminally truncated versions thereof lacking the F-box (∆F-box) were assessed for the ability to co-immunoprecipitate HA-RIPK3. Flag-Ank1, Flag-Ank1∆F-box, Flag-Ank6, and Flag-Ank6∆F-box comparably bound and reduced cellular levels of HA-RIPK3 (Figure 1E,F). Thus, Ank1 and Ank6 are capable of binding to RIPK3 and reducing its cellular levels though not as robustly as vIRD. Additionally, Ank1 and Ank6 each reduce RIPK3 levels in an F-box-independent manner.

### 3.2. RIPK3 Levels Are Lower in O. tsutsugamushi-Infected Cells

**Figure 2:**
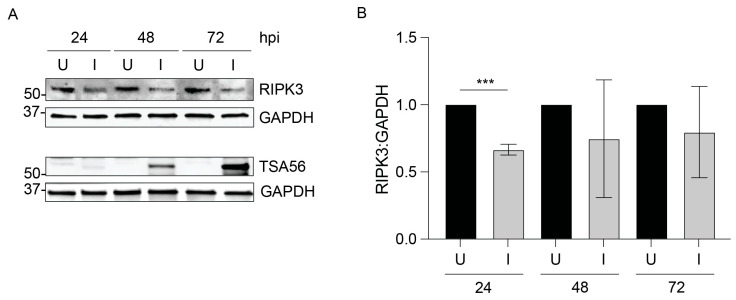
RIPK3 levels are decreased in *O. tsutsugamushi*-infected endothelial cells. EA.hy926 cells were mock-infected (U) or infected (I) with *O. tsutsugamushi*. (**A**) Whole-cell lysates harvested at 24, 48, and 72 hpi were analyzed by immunoblot with antibodies against RIPK3, GAPDH, and *O. tsutsugamushi* TSA56. (**B**) The RIPK3:GAPDH densitometric signal ratio per sample was calculated and normalized to the respective uninfected condition per timepoint. Data are presented as mean ± standard deviation of three independent experiments. Student’s *t*-test was used to test for significant differences between uninfected and infected conditions at each timepoint. Statistically significant values are indicated as *** *p* < 0.001.

Given that *O. tsutsugamushi* expresses *ank1* and *ank6* throughout infection of host cells [23], we assessed if the pathogen lowers RIPK3 levels. HeLa cells normally do not express RIPK3 and therefore could not be used for this purpose [44]. Human EA.hy926 endothelial cells, which are a proven model for studying *Orientia*-host cell interactions [26,36], were synchronously infected and monitored by immunoblot analysis over 72 h. Infection was confirmed by detection of the *Orientia* outer membrane protein, TSA56 [40] (Figure 2). RIPK3 levels trended lower at all timepoints in infected cells and were significantly reduced at 24 hpi. Thus, *O. tsutsugamushi* reduces RIPK3 levels during infection.

### 3.3. O. tsutsugamushi Fails to Inhibit Necroptosis Induced by RIPK3 Overexpression

**Figure 3:**
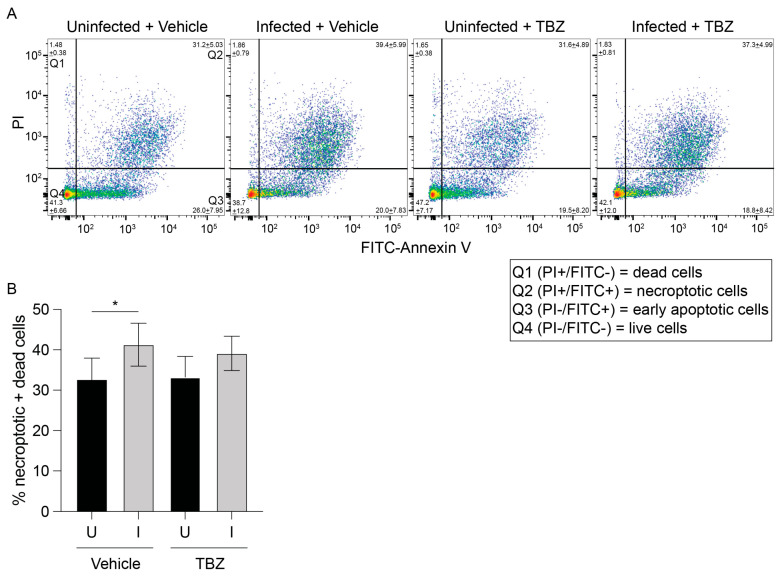
Uninfected and infected EA.hy926 cells resist TBZ-induced necroptosis. EA.hy926 cells were mock-infected (U) or infected with *O. tsutsugamushi* (I). At 72 hpi, cultures were treated with vehicle (Veh) or TBZ (1 h pretreatment with 1 μM BV6 + 20 μM zVAD-fmk followed by 10 ng mL^−1^ TNF for 4 h). Cells were then incubated with PI and FITC-Annexin V and analyzed by flow cytometry. The mean percentage of cells per quadrant ± one standard deviation was calculated and depicted in representative dot plots (**A**). Quadrant 1 (Q1; FITC-/PI+) and Q2 (FITC+/PI+) cells are necroptotic and dead, respectively. Q3 (FITC+/PI-) cells are early apoptotic. Q4 (FITC-/PI-) cells are alive. The combined percentages of dead and necroptotic (Q1 and Q2) cells per sample are displayed in (**B**). Data in (**B**) are presented as mean ± standard deviation values of six experiments. One-way ANOVA with Tukey’s post hoc test was used to test for significant differences between all samples. Statistically significant values are indicated as * *p* < 0.05.

We next sought to examine if necroptosis occurs in *O. tsutsugamushi*-infected cells. Necroptosis can be pharmacologically induced using a combination of a Smac mimetic (BV6) and pan-caspase inhibitor (zVAD-fmk) to inhibit apoptosis followed by the addition of TNF (TBZ) [45]. *O. tsutsugamushi*-infected and mock-infected EA.hy926 cells were treated with TBZ or vehicle at 72 hpi, incubated with Annexin V and propidium iodide (PI), and analyzed by flow cytometry. Annexin V binds phosphatidylserine when exposed on the plasma membrane’s outer leaflet during cell death. PI is a DNA-binding dye that crosses plasma membranes of dying cells. The combined percentage of dead (PI-single positive) and dying (annexin-V and PI-dual positive) cells did not appreciably change between vehicle- and TBZ-treated uninfected or infected cells (Figure 3). Moreover, the modest increase in dead/dying cells between uninfected and infected cells was comparable in the presence and absence of TBZ. This was likely due to apoptosis that had occurred by 72 hpi prior to the addition of BV6 and zVAD-fmk [23]. Thus, EA.hy926 cells are relatively insensitive to TBZ whether infected with *O. tsutsugamushi* or not.

**Figure 4:**
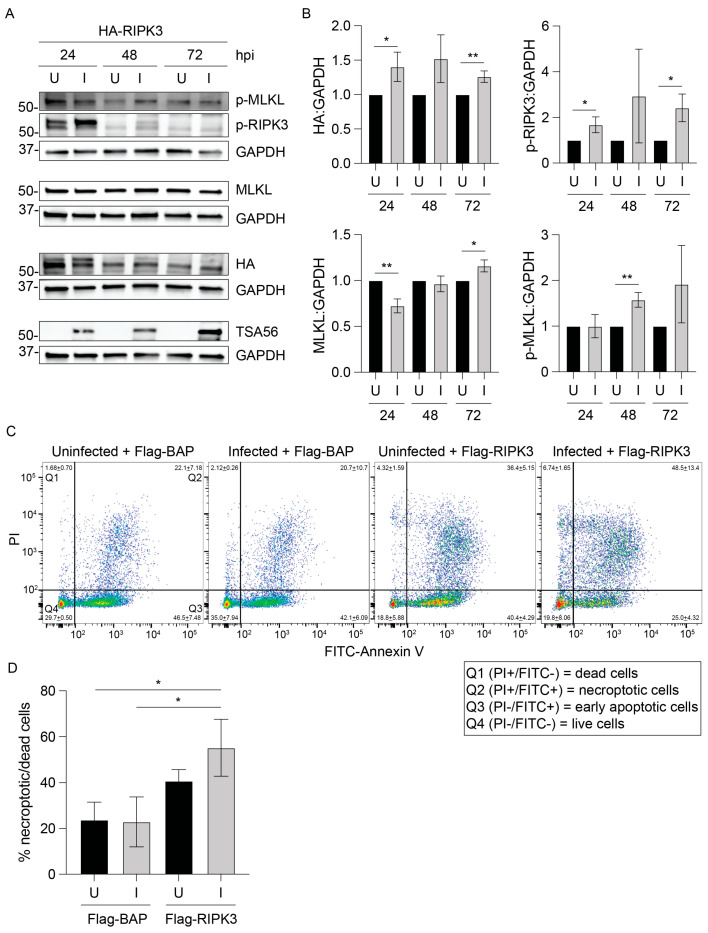
*O. tsutsugamushi* sensitizes HeLa cells to necroptosis induced by RIPK3 overexpression. (**A**,**B**) HeLa cells were transfected to express HA-RIPK3 for 16–24 h then mock-infected (U) or infected with *O. tsutsugamushi* (I). Whole-cell lysates harvested at 24, 48, and 72 hpi were analyzed by immunoblot (**A**) with antibodies against HA, phosphorylated (p)-RIPK3, MLKL, p-MLKL, TSA56, and GAPDH. (**B**) Densitometric signals of HA, p-RIPK3, MLKL, and p-MLKL normalized to GAPDH were calculated per sample and normalized to the corresponding uninfected sample at each timepoint. (**C**,**D**) U and I HeLa cells were transfected to express Flag-BAP or Flag-RIPK3 at 24 hpi. At 48 hpi, cells were incubated with PI and FITC-Annexin V and analyzed by flow cytometry. The mean percentage of cells per quadrant ± one standard deviation was calculated and depicted in representative dot plots (**C**). Q1 (FITC-/PI+) and Q2 (FITC+/PI+) cells are necroptotic and dead, respectively. Q3 (FITC+/PI-) cells are early apoptotic. Q4 (FITC-/PI-) cells are viable. The combined percentages of dead and necroptotic (Q1 and Q2) cells per sample are displayed in (**D**). Results are representative of three independent experiments. Data are presented as mean ± standard deviation (**B**,**D**). Student’s t-test was used to test for a significant difference between two samples (**B**). One-way ANOVA with Tukey’s post hoc test was used to test for significant differences between all samples (**D**). Statistically significant values are indicated as * *p* < 0.05, ** *p* < 0.01.

HeLa cells can be sensitized to necroptosis by transfecting them to ectopically express RIPK3 [44]. Therefore, as an alternative method for studying necroptosis during *O. tsutsugamushi* infection, HeLa cells were transfected to express HA-RIPK3 and infected 24 h later. HA-RIPK3 and phosphorylated RIPK3 were detected at all timepoints, although levels were most robust at 24 hpi (Figure 4A,B). Both proteins were observed as doublets, particularly in infected samples. This is likely because activated RIPK3 is both phosphorylated and K63-ubiquitinated, which promotes its association with the necrosome [46]. Both the higher-molecular-weight (ubiquitinated) and lower-molecular-weight (non-ubiquitinated) RIPK3 forms were detected by HA and p-RIPK3 antibodies and thus were included in the densitometric analyses. The observed declines in HA-RIPK3 and phospho-RIPK3 at 48 and 72 hpi (Figure 4A), which correlate with 72 and 96 h post-transfection, respectively, are likely due to gradual loss of the plasmid encoding HA-RIPK3 and/or necroptosis. Nonetheless, both phosphorylated and non-phosphorylated RIPK3 forms were elevated in infected cells (Figure 4A,B). Endogenous MLKL was present in uninfected and infected cells throughout the time course, and phosphorylated MLKL levels were higher in infected cells. These data indicate that ectopic overexpression of RIPK3 promotes necroptotic signaling that *O. tsutsugamushi* is unable to inhibit.

As a complementary approach, *O. tsutsugamushi*-infected HeLa cells were transfected at 24 hpi to express Flag-RIPK3 or Flag-BAP and assessed by flow cytometry 24 h later (48 hpi) when bacterial intracellular growth is logarithmic [36]. The combined percentage of PI single-positive and Annexin V/PI dual-positive cells between uninfected and infected cells expressing Flag-BAP was unchanged (Figure 4C,D), which is consistent with *Orientia*-infected cells being recalcitrant to PCD prior to 72 hpi [20,21,23,24]. The percentage of dead/dying cells was 2.5-fold higher in infected cells expressing Flag-RIPK3 versus Flag-BAP. Hence, the inability of *O. tsutsugamushi* to inhibit the RIPK3-MLKL signaling axis results in PCD.

### 3.4. MLKL Does Not Associate with Intracytosolic O. tsutsugamushi

**Figure 5:**
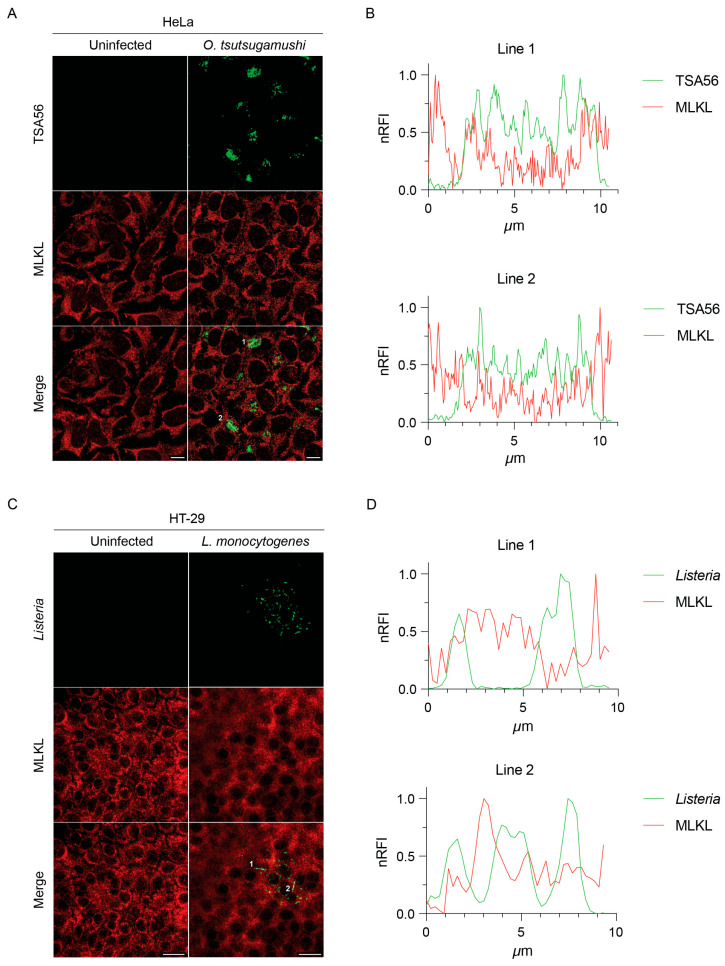
Endogenous MLKL does not co-localize with *O. tsutsugamushi* or *L. monocytogenes. O. tsutsugamushi*-infected HeLa cells (**A**,**B**), *L. monocytogenes*-infected HT-29 cells (**C**,**D**), and uninfected controls were fixed in ice-cold methanol and immunolabeled with antibodies against MLKL (**A**,**C**), *O. tsutsugamushi* TSA56 (**A**), and *L. monocytogenes* (**C**) and examined by immunofluorescence microscopy. Representative immunofluorescence micrographs are presented in panels (**A**,**C**). (**B**,**D**) Relative fluorescence intensity plots of green and red pixels from immunofluorescence micrographs of *O. tsutsugamushi* (**A**) or *L. monocytogenes*-infected (**C**) cells normalized to the highest fluorescence intensity per channel moving left to right along the white lines. Scale bars = 10 μm (**A**) or 20 μm (**C**). Data are representative of three independent experiments.

The RIPK3-MLKL pathway also executes intrinsic functions separate from PCD [42,47,48]. For instance, activation of this pathway in HT-29 intestinal epithelial cells by the Gram-positive foodborne pathogen, *Listeria monocytogenes*, suppresses *Listeria* intracellular growth in a manner independent of necroptosis. This phenomenon was linked to MLKL predicated on the abilities of recombinant MLKL to bind and potentially disrupt the plasma membrane of *Listeria* in cell-free assays and ectopically expressed MLKL-Flag to robustly colocalize with the bacterium in HT-29 cells in the absence of TBZ stimulation [49]. To determine if endogenous MLKL associates with *O. tsutsugamushi*, synchronously infected HeLa cells were fixed, immunolabeled for MLKL and TSA56, and visualized using immunofluorescence microscopy. In parallel, HT-29 cells infected with *L. monocytogenes* were also examined. Despite pronounced detection of MLKL in both sets of infected cells, we did not observe colocalization of it with either intracytosolic bacterial population (Figure 5). Hence, endogenous MLKL does not associate with *O. tsutsugamushi* and does not recapitulate colocalization with *L. monocytogenes* reported for ectopically overexpressed MLKL.

## 4. Discussion

Necroptosis is a proinflammatory PCD pathway that serves as a key line of innate defense against intracellular microbes [1,50]. As an obligate intracellular pathogen that grows to a high load in the cytosol, *O. tsutsugamushi* would have been under selective pressure in its co-evolutionary relationship with eukaryotic host cells to stealthily replicate without eliciting PCD. Indeed, *Orientia* avoids stimulating and inhibits apoptosis until late in infection [20,21,23,24]. Here, we extend this stealth strategy to necroptosis by showing that *Orientia* reduces RIPK3 cellular levels and does not elicit PCD in cells overexpressing BAP. However, the bacterium fails to suppress RIPK3 and MLKL phosphorylation and death in cells overexpressing RIPK3. We were particularly interested in assessing necroptosis during *O. tsutsugamushi* infection due to its pro-inflammatory nature. Necroptotic cells drive a systemic inflammatory response and major organ damage through the release of DAMPs and inflammatory cytokines and chemokines such as IL-1α/β, IL-6, IL-33, and CCL2 [42,51]. *O. tsutsugamushi*-infected mice and severely ill scrub typhus patients experience widespread vasculitis and major organ damage secondary to a disseminated inflammatory response characterized by secretion of these and other cytokines, suggesting that necroptosis may contribute to the pathobiology of *O. tsutsugamushi* infection [52,53,54,55]. Notably, various bacterial pathogens, including *Staphylococcus aureus*, *Streptococcus pneumoniae*, *Mycobacterium tuberculosis*, and *Salmonella typhimurium*, permit or even induce necroptosis to facilitate their dissemination or kill immune cells [50]. As *O. tsutsugamushi* sensitizes cells to necroptosis, the increased TNF levels in symptomatic scrub typhus may induce necroptosis of infected cells, which could promote bacterial spread [54]. Further studies will be needed to determine precisely how necroptosis benefits or harms *O. tsutsugamushi* during infection. A valuable tool will be RIPK3-deficient mice, which have been employed to discern the contributions of RIPK3 and necroptotic signaling to the pathobiology of other microbial diseases [4,49,56,57,58,59,60,61].

*O. tsutsugamushi* reduction of RIPK3 levels presumably involves Ank1 and Ank6 given that both phenocopy this effect when ectopically expressed. Although these Anks share a bipartite AR-F-box architecture with cowpox vIRD and all three proteins similarly nucleate the SCF complex and inhibit NF-κB nuclear accumulation [27,28,62], we discovered that Ank1 and Ank6 are mechanistically different from vIRD with respect to RIPK3 binding and degradation. The relatively low efficiencies by which Ank1 and Ank6 bind and reduce cellular levels of RIPK3 compared to vIRD correlate with the inability of *O. tsutsugamushi* to deplete RIPK3 levels or inhibit induced necroptosis, unlike cowpox virus [4]. One possible explanation for these functional differences lies in the structure of their AR domains. vIRD contains six ARs spaced in pairs [62], while Ank1 and Ank6 each carry four evenly spaced ARs [27]. Since RIPK3 interacts with vIRD at the AR domain [4], it is possible that the loss of two ARs or the overall difference in structure is sufficient to alter the three-dimensional positioning of RIPK3 binding to Ank1 and Ank6 such that the SCF complex can no longer efficiently ubiquitinate RIPK3. An additional contributing factor could be differences in the Ank1 and Ank6 AR residues that mediate RIPK3 binding resulting in lower affinities. We cannot rule out the possibility that our co-immunoprecipitation experiment detected HA-RIPK3 bound to Flag-Ank1 or Flag-Ank6 as part of a larger complex that sterically blocked SCF-mediated polyubiquitination of RIPK3. Nonetheless, Ank1 and Ank6 still significantly lower RIPK3 levels by undefined mechanisms. Both can translocate into the nucleus [27,28], which could position them to interfere with RIPK3 expression.

Since *O. tsutsugamushi* does not impede necroptosis, it might gain advantages independent of PCD by targeting RIPK3. For instance, RIPK3 promotes inflammatory cytokine production independent of MLKL activation or necroptosis through activating the NLRP3 inflammasome [58,63,64]. It promotes cell cycle progression and regulates NF-κB activity [47,65,66], the latter of which *Orientia* inhibits [27,34]. RIPK3 induces reactive oxygen-species generation and modulates central metabolism, glutamine catabolism, and mitochondrial fission [67,68]. *Orientia* impairs host-cell metabolic activity in a mitochondrial-dependent manner via an unknown mechanism with amino acids serving as the primary nutrient source that it parasitizes [36]. RIPK3 also promotes autophagic flux and phosphorylates Ulk1 to activate Atg5-independent autophagy [69,70]. *O. tsutsugamushi* activates autophagy upon infection but ultimately evades xenophagic clearance and returns autophagy to baseline by 6 to 24 hpi [71,72]. By lowering RIPK3 levels, *Orientia* could modulate any of these non-necroptotic antimicrobial pathways. MLKL has been proposed to associate with and inhibit *L. monocytogenes* intracellular growth based on observations obtained using ectopically expressed MLKL [49]. Yet, we found that endogenous MLKL does not colocalize with *O. tsutsugamushi* or *L. monocytogenes*. Importantly, the specificity of the MLKL antibody used herein, which was not available at the time the original study was performed [49], has been independently validated [73]. Moreover, the previous and current *Listeria* experiments were performed with HT-29 cells [49]. While we cannot exclude the possibility that the reported differences in MLKL-*Listeria* colocalization are due to the use of distinct *L. monocytogenes* strains [49], we respectfully submit that it most likely reflects differential colocalization of ectopically overexpressed versus endogenous MLKL.

Most *O. tsutsugamushi* experiments herein were conducted using HeLa cells, which are useful models for studying *Orientia* infection and Ank effector pathobiology, as well as investigating the actions of ectopically expressed RIPK3 in necroptosis [23,26,27,28,33,34,36,38,40,74,75,76,77,78,79,80,81,82,83]. The insensitivity of EA.hy926 endothelial cells to TBZ and *Orientia* stimulation of necroptosis is consistent with previous reports that this PCD pathway is limited in endothelial cells [84,85]. It will be important to determine if the results in this report extend to other cell types that *O. tsutsugamushi* infects in vivo, such as dendritic cells and macrophages.

In conclusion, this study provides a first step toward understanding the interplay of *O. tsutsugamushi* and necroptosis. Despite having similar Ank arsenals that modulate NF-κB, *Orientia* and cowpox Anks differentially target RIPK3 [27,28,62]. Unlike cowpox, which eliminates RIPK3 altogether to prevent necroptosis [4], *O. tsutsugamushi* lowers RIPK3 levels and does not trigger necroptotic cell death. However, in cells ectopically expressing RIPK3 it is apparent that *Orientia* sensitizes the host cell to and is unable to inhibit necroptosis. Short-term future work is needed to determine if *O. tsutsugamushi* infection of other cell types elicits necroptosis and if the bacterium’s modulation of RIPK3 cellular levels counters necroptosis-independent host cell processes. Longer-term future work should assess if *Orientia* sensitization of host cells to necroptosis contributes to the systemic inflammation seen in severe scrub typhus.

## Data Availability

The original contributions presented in this study are included in the article. Further inquiries can be directed to the corresponding author.

## Abbreviations

The following abbreviations are used in this manuscript:

PCD	Programmed cell death
RIPK1	Serine/threonine kinase receptor interacting protein 1
RIPK3	Serine/threonine kinase receptor interacting protein 3
MLKL	Mixed-lineage kinase domain-like
AR	Ankyrin repeat
vIRD	Viral inducer of RIPK3 degradation
SCF	Skp1/cullin-1/F-box
NF-κB	Nuclear factor kappa-B
hpi	Hours post infection
MOI	Multiplicity of infection
TSA56	56 kDa type-specific antigen
HA	Hemagglutinin
BAP	Bacterial alkaline phosphatase
DPBS	Dulbecco’s phosphate-buffered saline
EDTA	Ethylenediaminetetraacetic acid
BHI	Brain heart infusion
OD	Optical density
PCR	Polymerase chain reaction
DNA	Deoxyribonucleic acid
PBS	Phosphate-buffered saline
TBS	Tris-buffered saline
RIPA	Radioimmunoprecipitation assay
NP-40	Nonidet P-40
SDS-PAGE	Sodium deoxycholate–polyacrylamide gel electrophoresis
GAPDH	Glyceraldehyde-3-phosphate dehydrogenase
HRP	Horseradish peroxidase
TBZ	TNF + BV6 + zVAD-fmk
TNF	Tumor necrosis factor
BSA	Bovine serum albumin
PI	Propidium iodide
ANOVA	Analysis of variance
DAMP	Damage-associated molecular pattern
IL-1	Interleukin-1
IL-6	Interleukin-6
IL-33	Interleukin-33
CCL2	C-C motif chemokine ligand 2
NLRP3	NOD-, LRR- and pyrin-domain-containing protein 3
Ulk1	Unc-51-like autophagy activating kinase 1
Atg5	Autophagy-related 5
